# Enhancing patient-centred care in dentistry: a narrative review

**DOI:** 10.1093/bmb/ldad026

**Published:** 2023-10-13

**Authors:** Camilla Böhme Kristensen, Koula Asimakopoulou, Sasha Scambler

**Affiliations:** Faculty of Dentistry Oral & Craniofacial Sciences, Centre for Host-Microbiome Interactions, King’s College London, London SE1 9RW, UK; Visiting Professor of Health Psychology, Faculty of Dentistry Oral & Craniofacial Sciences, Centre for Host-Microbiome Interactions, King's College London, London SE1 9RW, UK; Faculty of Dentistry Oral & Craniofacial Sciences, Centre for Host-Microbiome Interactions, King’s College London, London SE1 9RW, UK

**Keywords:** patient-centred care, dentistry, healthcare, theoretical models, implementation research

## Abstract

**Introduction:**

Patient-centred care (PCC) is widely used within the medical setting, but there is a need for more research on PCC implementation in dentistry.

**Sources of data:**

A narrative review was conducted with literature identified from the Ovid Interface, including several databases such as Embase and Medline.

**Areas of agreement:**

PCC is associated with better health outcomes for patients, and greater work satisfaction among healthcare professionals.

**Areas of controversy:**

Efforts to implement PCC in dentistry are lacking due to several issues including non-consensus about PCC definition and lack of explicit guidelines on how to implement PCC in dentistry.

**Growing points and areas timely for developing research:**

A theory-derived model of PCC explicitly designed for the dental setting was identified. This serves as a starting point to enhance PCC in dentistry, though further research is needed to empirically test the implementation of this model.

## Patient-centred care

Patient-centred care (PCC) is a term used freely by professionals in the healthcare sector^1^ and has been defined as ‘care that is respectful of and responsive to individual patient preferences, needs and values, and ensuring that patient values guide all clinical decisions’.^2^ Gerteis *et al.*^3^ further propose that PCC entails eight dimensions including patient preferences; information and education; access to care; emotional support; family and friends; continuity and transition; physical comfort and coordination of care. PCC is widely used in the field of medicine and adopted and endorsed by the National Institute for Health and Care Excellence (NICE).^1^ It is thus a holistic approach to care that considers the biopsychosocial factors of the patient during the treatment process.

Individual biological and genetic factors may account for one-third of all determinants of health.^4^ Other determinants of health include behavioural patterns (40%), social circumstances (15%), environmental exposure (5%) and healthcare (10%).^4^ Psychosocial factors are also central to oral health.^5^ For example, dental anxiety (a psychological factor)^6^ and socioeconomic status (a social factor)^7^ are key variables in understanding dental caries. Dental anxiety may be associated with avoidance of dental treatment and help-seeking,^6^ while costs of dental treatment may be a barrier to seeking care among individuals of low socio-economic status.^7^ It is thus imperative to consider all patient factors (e.g. psychosocial factors) and circumstances to maintain and improve health, which is what a patient-centred approach can offer. PCC has several benefits to the patient, with research suggesting that PCC leads to improved health outcomes; reduced use of care; enhanced patient satisfaction and enhanced health status.^8–10^ It also offers benefits to healthcare professionals, including reduced levels of litigation and greater work satisfaction.^11^ Such outcomes are exceptionally beneficial for patients and healthcare professionals alike, fully justifying the enthusiasm for delivering patient-centred healthcare.^1^

### A review of issues with PCC in dentistry

While PCC is widely adopted in the field of medicine,^12^ efforts to adopt and implement the principles of PCC have also been attempted within the dental field. However, this has proven difficult due to several issues.

### Definitions of PCC

First, it appears from systematic review evidence that there is no shared definition of what PCC entails within the dental literature. According to Scambler and Asimakopoulou^13^ and Mills et al.,^1^ this has implications for implementing effective PCC in practice. Scambler *et al.*^14^ conducted a systematic search to explore how PCC was defined within the dental literature. Of the 390 identified articles, 28 were deemed eligible for synthesis. In over half of the articles, the definitions of PCC reflected good practice, rather than the principles of PCC. Moreover, many of the identified articles referred to PCC in terms of providing care that is holistic and person-focused, while others defined PCC as respecting the patient’s decisions; making the patient feel good about the treatment they are receiving/going to receive; ensuring effective communication from the dental professional or having a flexible approach to decision-making.

Several papers identified in the work by Scambler and Asimakopoulou^14^ also discussed the need for PCC in dentistry, specifically addressing the need for patients to be provided with information about their rights to be involved in the decision-making about their care. Yet, only three papers explicitly involved patient decision-making and provided them with the resources necessary to make such decisions. Similar findings arise from a separate systematic review by Mills *et al.*.^1^ Here, the authors too set out to conduct a systematic search to ascertain whether a shared understanding of PCC had been achieved in dentistry. A total of 85 articles were identified of which 4 were included for synthesis. Based on their review, it was found that ‘there is presently inadequate evidence available to understand PCC within general dentistry, let alone measure it’ and that ‘further research is needed to understand the key features of PCC in dentistry’.^1^

### Shared decision-making

A related issue is concerning shared decision-making (SDM).^1–3^ SDM is defined by the NICE guidelines as: ‘a collaborative process that involves a person and their healthcare professional working together to reach a joint decision about their care’.^15^ It is a key aspect of PCC,^16^^,^^17^ as it is no longer acceptable for any healthcare professionals, including those of the dental profession to make decisions about patient care, without the complete involvement of the patient.^18^ Informed consent is central in SDM, and provides the patient with the autonomy to actively make decisions about their care. The provision of informed consent may contribute to a reduction of patient confusion about treatment and prevent unexpected medical disputes.^19^

While the importance of SDM is reflected in several dental schools^20^^,^^21^; evidence suggests that PCC and/or SDM are not incorporated into dental teaching routinely.^22^ Learning PCC in practice is also limited. For example, evidence from a qualitative study indicated that few dental professionals learned PCC from observing senior colleagues, while most stated that they had no formal training on PCC, and how it should be practised.^22^ The lack of education and training on PCC may pose limitations to the knowledge, awareness and skills of PCC among newly qualified dental professionals. This may thus inhibit the implementation of PCC in dentistry moving forward.

Though the concept of SDM is well documented in the literature,^23^ it has no universally accepted definition in healthcare, and it cannot be measured.^24^ This issue was demonstrated in a recent qualitative study, which explored dental professionals’ views on SDM.^23^ It was found that dental professionals supported SDM in theory, however, there were connotations of defensive practice. Defensive practice is defined as ‘practice that is deliberately chosen to protect the professional worker, at the possible expense of the well-being of the client’.^25^ Dental professionals are involved with orthodontic and endodontic treatments, which have the potential to damage the patient and/or be life-threatening.^26^^,^^27^ Moreover, D’Cruz (2016) argues that dental professionals ‘gamble that the patient is sufficiently motivated to act on preventive advice… If the dental professional gets it wrong, the patient’s condition may worsen’.^28^ Therefore, defensive practice may be an issue among dental practitioners in the UK, citing fear about the risks associated with treatment on the patient’s health and wellbeing, and the potential impact of patient non-adherence to treatment. Defensive practice thus deters true SDM and PCC^23^ because it fails to incorporate collaboration between the dental professional and patient. Hayer and Wassif (2022) further noted how defensive practice may happen due to a lack of understanding of what SDM entails.^23^ Nonagreement among dental professionals about the SDM definition makes it difficult to implement it in care.^24^ Furthermore, discrepancies between ‘what patients can expect from their healthcare experience versus what and how this experience can be delivered by the practitioner’ are also barriers to adopting SDM in dentistry according to Hayer and Wassif.^23^ Keeping in mind the positive impact of PCC,^8–10^ defensive practice and thus non-person-centred care may adversely affect the healthcare system and result in reduced uptake of care and patient satisfaction, while consequences for the healthcare professionals may include reduced work satisfaction.

### Guidelines, PCC models and the healthcare system

The second issue with PCC practice in dentistry is that there are no explicit guidelines on how to implement PCC in dentistry.^13^ While The UK General Dental Council (GDC) promotes PCC through principles of patient respect, dignity and choices, it does not explicitly offer details on how to achieve this form of care. For example, according to Scambler and Asimakopoulou, the GDC does not discern between different contexts of care and different types of dental professionals, nor do they provide examples of how to implement PCC in clinical practice.^13^ These issues may prevent dental professionals who wish to promote PCC from doing so.

Correspondingly, there are issues with adopting PCC models from medicine into the dental setting.^18^ Although physicians and dental professionals are expected to care for their patient’s health,^12^ there are several differences in care between the two fields. For instance, according to work by Freukel and Lurie,^29^ in medicine, there are often multidisciplinary teams and shared responsibilities between different health professionals, whereas in dentistry, the responsibility is imposed solely on the dental professional, who may also be responsible for correcting work done by others. Freukel and Lurie^29^ furthermore argue that medicine and dentistry have different rules of ethics, which makes the care between the two areas different.^29^ In terms of patient needs, there are too obvious differences between medicine and dentistry. For example, studies in medicine found that patients value communication, partnership and health promotion,^20^ whereas dental patients value roles in decision-making, confidence and trust in the dental professional’s knowledge.^30^ Recent efforts have, however, been made to address the issues around the implementation of PCC in dentistry.^31^ This is a step in the right direction.^18^ However, Gardner^32^ and Mills *et al.*^18^ argue that commissioners should consider the unique aspects of dentistry and that any new material should not rely on PCC models developed in medicine, because they do not easily translate into dentistry.

Lastly, the influence of the healthcare system can impact PCC delivery. The healthcare system is complex and involves several actors at different institutional levels. The different actors within the healthcare system may have different needs, conditions and interests. This makes implementing PCC into the healthcare system difficult and highlights why a multi-perspective approach is required to incorporate all ‘wants and needs’.^33^

### Current PCC models in dentistry

According to a recent commentary by Mills *et al.*,^18^ there are six current models of PCC developed for dentistry.^34–39^ Mills *et al.*^18^ noted that all the models consider the patient’s social context, and how they understand the presenting disease. Two of the models^34^^,^^36^ were developed based on evidence from patients’ perspectives, and views from the patient and the dental professionals. SDM was noted in four models,^34^^,^^35^^,3839^ while two of the models^34^^,^^40^ noted the importance of the broader healthcare context. Mills *et al.*^18^ summarized that generally speaking, the existing PCC models in dentistry note the importance of compassionate care and accentuate patient relationship-building. However, the authors also argued that most of the models were developed by and addressed to specific groups of patients and dental professionals who are not representative of the average patient/dentist population.

### Developing a PCC model in dentistry

**Fig. 1:**
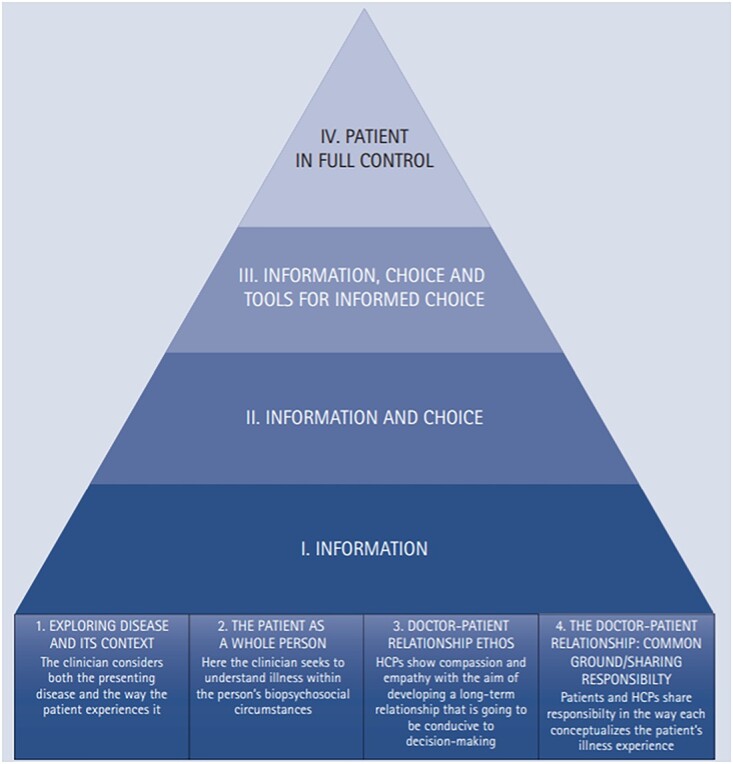
An emiprical model of PCC designed for dentistry. The model is derived from previous work by Scambler and Asimakopoulou (2014).

To address the issues discussed in this review, Scambler and Asimakopoulou^13^ developed a theory and empirically derived model of PCC. This model serves as a practical guide on how to implement PCC in dental clinics and aims to propose a uniform definition of what PCC entails. The model consists of four parts, which represent the core aspects of PCC, with an incorporated hierarchy of information and choice (Fig. 1).

#### Part 1: Exploring disease and the context in which it occurs

The first part of the model describes how dental professionals should consider how the symptoms and disease affect the patient’s life and circumstances. Although considerations of the symptoms are necessary, these should be considered in a wider context. For example, if a patient needs a front tooth extracted, the consultation should go beyond the technical aspects of the extraction itself. The dental professional should explore how the extraction impacts the patient’s overall life, including the need for a follow-up appointment and potential costs associated with that; the psychological impact (e.g. worries about appearance because of a missing front tooth); the social impact (e.g. difficulties with speech after tooth extraction, which may harm social relations); issues around taking pain-control medication with other medications the patient may take and the need for the patient to have potential children looked after by someone else, while he/she is receiving treatment/is recovering. The dental professional should thus consider any barriers that may hinder successful treatment, and discuss with the patient how such barriers might be overcome.

#### Part 2: The patient as a whole person

This part somewhat relates to Part 1: ‘Exploring disease and the context in which it occurs’. However, Part 2 focuses more on the patient and his/her biopsychosocial conditions. As an example, during a consultation where the aim is to improve oral hygiene using interdental cleaning tools, the consultation should be focused differently according to what type of patient is sitting in the chair. For example, a younger patient with adequate physical skills (e.g. manual dexterity or full eyesight) to engage with interdental cleaning requires a differently focused consultation compared with an older patient who may rely on others for help with oral hygiene.

Likewise, in a consultation where the objective is to reduce tooth decay in toddlers, the consultation should be focused differently depending on the parent/caretaker who is attending with their child. For instance, if the parent/caretaker is a single mum from a low-socioeconomic status with poor oral health herself, the consultation needs to be focused differently than to a parent/caregiver with more resources (e.g. financial resources) to support their child with toothbrushing. To summarize, the first two parts of the proposed PCC model suggest that the disease and/or presenting symptoms should be considered in the context of the biopsychosocial circumstances of the patient. This approach is in line with the NICE guidelines on patient practice in the NHS.^13^

Parts 3 and 4 of the model are centred on the relationship between the patient and the dental professional and describe the type of relationship needed for patients to make informed decisions about their oral health and care.

#### Part 3: The ethos of the healthcare professional-patient relationship

Part 3 describes the importance of the dental professional’s ability to be compassionate and empathetic. This is needed to form a long-lasting relationship with the patient to engage them in informed decision-making about their treatment. This process is in line with the NICE guidance, which promotes the need to treat patients with kindness, dignity, compassion, respect, courtesy, honesty and understanding.^12^ Part 3 of this model thus promotes the principles of good quality patient care, instead of the principles of PCC explicitly. However, the ethics of good quality patient care are necessary aspects of PCC.^13^

#### Part 4: The doctor–patient relationship

The final part describes how the dental professional and patient reach a mutual agreement in relation to the following three areas: (i) problem definition; (ii) determining the goals and priorities of the treatment and (iii) clarifying the roles of the dental professional and the patient, respectively. The first agenda point is to reach a shared understanding of the presenting oral health issues. If there are divergences or disagreements, consensus should be achieved prior to proceeding to agenda point two in the process of establishing a doctor–patient relationship. For instance, if the patient is expecting a treatment, which is not the most appropriate one based on the perspective of the dental professional, a discussion should be held to achieve treatment consensus. It is further imperative at this stage that the dentist provides the patient with adequate knowledge that will facilitate the patient’s ability to make an informed treatment decision.

### Challenges with maintaining clinical autonomy

The last part of PCC, which sets out to reach common grounds between the patient and the dental professional, is the most difficult aspect.^13^ For example, it is challenging to provide true PCC in the clinical setting, when there are discrepancies between what the patient wants in terms of treatment and what is clinically appropriate (and safe) from a professional perspective. Therefore, issues around clinical and patient autonomy can arise. Furthermore, it is argued whether it is ethical to give patients the illusion that they are involved in decision-making about their treatment when the only choice is to do what the dental professional advises.^13^ It is, however, important to note that PCC does not advocate unsafe treatment, regardless of what the patient wants. Nevertheless, these issues raise questions about whether the ethos of PCC can be truly applied in practice. For example, what happens in situations where treatment consensus cannot be reached? How does the dental professional know that they have provided the patient with enough information so he/she can make an informed choice about their treatment? Such issues around information sharing highlight the need for further guidance on how to truly implement PCC in dentistry.

### A hierarchy of information and choice

To guide how to achieve true PCC in dentistry, a hierarchy of information and choice was added to the model proposed by Scambler and Asimakopoulou. The hierarchy has the foundations of PCC at the bottom, which was outlined previously. On top of the foundational components of PCC are four levels: (i) information, (ii) information and choice, (iii) information, choice and tools for informed choice and (iv) patient in full control. The hierarchy suggests that clinical consultations are more patient-centred the higher they sit in the pyramid.

#### Level 1: Information

Level 1 describes the bottom level of the hierarchy. Here, the patient is provided with relevant information about their health. This level describes what usually occurs in routine consultations, whether they are PCC-focused or not. Information provided should be evidence-based and given at the most basic level to ensure comprehension. For instance, it would be expected that the dental professional describes what gingivitis is, while providing information about risks and relevant self-care activities the patient can do to treat the condition.

#### Level 2: Information plus choice

This level is approaching the ethos of PCC. Here, the patient is presented with potential choices of treatment alternatives, in addition to the procedure described in Level 1. If there are no treatment alternatives, the dental professional and the patient should explore the single available treatment versus the non-treatment alternative. Using the example gingivitis scenario, Level 2 would entail that the dental professional discuss the various self-management behaviours the patient can do to treat the gingivitis. A discussion of the possible consequences of consciously not performing self-care behaviours should also be facilitated. This type of care differs from Level 1 because the patient is advised that they have a choice in how they choose to manage the gingivitis. In Level 1, however, it is assumed that the patient will want to follow the self-care behaviours advised by the professional. Thus, Level 2 facilitates a choice for the patient in terms of deciding what the next steps should be (or not be). It is important to note that it is not proposed that patients are encouraged to disregard medical advice. However, the premise is that the dental professional understands that the patient has a choice in their care.

#### Level 3: Information, choice plus tools for informed choice

An additional component of care is added at Level 3. Here, the dental professional should provide support that allows the patient to make an informed choice about their care. Thus, all treatment options should be introduced to the patient, whilst considering the psychosocial aspects of the patient. At this level, the dental professional goes beyond acknowledging that the patient has a treatment choice, which is different from Level 2. Instead, the consultation is expanded in that the professional helps the patient explore various alternatives, whilst keeping in mind the psychosocial context of the patient.

#### Level 4: The patient is in full control of their care

At Level 4, the patient should have been provided with the information, choice and tools to make an informed treatment decision that considers their biopsychosocial aspects. The patient-initiated choice of treatment may be conflicting with the dental professional’s opinion. However, if the proposed process of PCC has been followed through extensively (with a specific focus on reaching treatment consensus), then the care can be described as meeting Level 4 of the PCC hierarchy. Although the patient's choice may go against the advice of the dental professional, it is important to comment that following the PCC steps outlined may result in the patient asking the professional to make a treatment decision for them. Therefore, if the treatment decision is based on the results of an informed, patient-focused process, the patient is in full control of their care. This also applies in circumstances where the patient chooses to follow clinical advice, and in situations where they choose not to. Level 4 is thus about supporting patients in deciding what they feel is the right course of action with their care.

## Discussion and implications

This review discussed the issues with PCC in dentistry and identified a theory-derived, dentistry-specific model of PCC. It offers a practical guide on how to implement PCC in dentistry to enhance care.^13^ PCC considers the biopsychosocial aspects of a patient and supports the patient with SDM in their care.^3^ Person-centred care has several positive outcomes for patients and healthcare professionals alike,^8–10^ making it an important practice within healthcare.^1^ However, although PCC is widely implemented and adopted by the wider UK health service,^12^ the dental field is lagging behind in adopting PCC due to two main issues. The first is a lack of consensus about what PCC and SDM mean in the dental literature. While some authors described PCC as a holistic approach to care, others described PCC as entailing patient choice about their treatment.^14^ Similarly, it was found that dental professionals supported SDM in theory; though, nonagreement on what SDM entails was also apparent.^23^ The lack of consensus in PCC/SDM definitions makes it difficult for dental professionals to implement patient-centredness care—because how does one implement a concept that is not clearly defined or agreed upon?

The second prominent issue related to the lack of explicit PCC guidelines for implementation, insufficient, dentistry-specific PCC models, and the lack of teaching and training on PCC in dental education. The implementation issues of PCC in dentistry thus force this profession to continue with standard, good practice, yet, this may not be enough. The prevention of oral disease and complications relies heavily on self-care behaviours such as tooth brushing, interdental cleaning and smoking cessation.^41^ Nevertheless, the dental field faces a difficult challenge with patient adherence to these self-care practices.^42^ This is evident from the large body of research suggesting that poor oral health is a global burden, with periodontal disease prevalence seen in approximately half of the UK adults, and tooth decay affecting one-third of the population.^43^^,^^44^ In addition to causing other physical health complications,^45^ poor oral health also affects a person’s quality of life and psychological wellbeing.^46^ Oral health is thus an important aspect of health and well-being. Citing the evidence that demonstrates the effectiveness and positive impact of PCC,^8^^,^^9^^,^^11^ it is problematic that this approach is not properly utilized in the dental field.

**Fig. 2:**
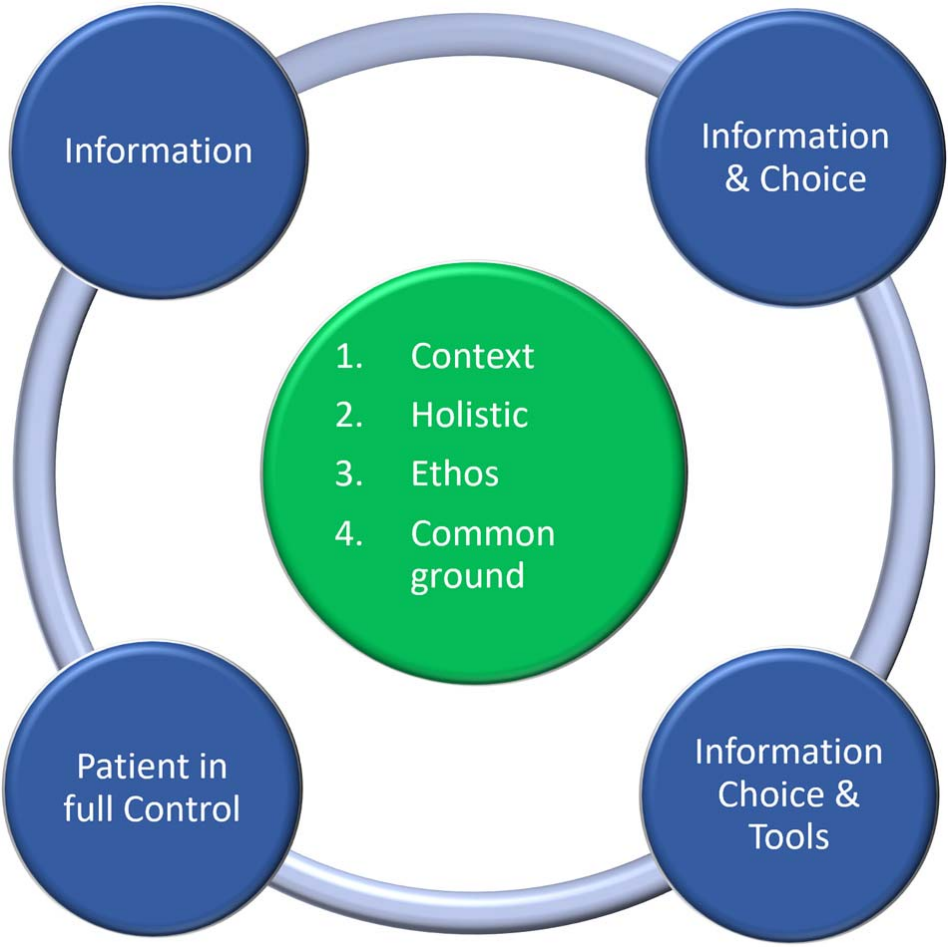
A reconceptualized model of PCC designed for dentistry. The model is derived from work by Scambler and Asimakopoulou (2014).

In summary, this paper identified a model of PCC tailored for dentistry.^13^ While this model is theory-derived, it is important to consider its utilization in light of the following limitations. For example, it may be that PCC is wider than information and choice as suggested in the model. Furthermore, reducing the model to information and choice may make the model more implementable, however, it may miss important aspects of PCC and thus, be less representative of true PCC. Moreover, the hierarchical structure of the model suggests that a higher level is better than a lower level. However, higher levels within the hierarchy are only better if the patient wants to be involved. The model could be reconceptualized into a circle instead to reflect different aspects of PCC, rather than levels (Fig. 2).

In conclusion, while the authors note that this model is yet to be empirically tested,^13^ it nevertheless offers a tool for reflection and can be used as a self-assessment method among dental professionals to understand what level of PCC the clinic is currently conducting. This paper serves as a call to action for clinicians, educators, regulators and commissioners in the dental field to implement PCC in teaching, training and clinical practice.

## Data availability

No new data were generated or analysed in support of this review.

## Conflict of interest

The authors declare no conflict of interest. 
